# Texas State Medical Association—Delinquent Members

**Published:** 1890-07

**Authors:** 


### TEXAS STATE MEDICAL ASSOCIATION.—DELINQUENT MEMBERS.

There are one hundred and ten members of the Association who have not paid their dues in three years; their names will not appear in the roll in the forthcoming Transactions, unless remittance of $10 be made prior to August 10, prox. There is an ordinance in force which allows such members to be reinstated upon the payment of $10 (the amount paid by new members). This $10 is in lieu of all arrears. The Secretary has sent notice to the one hundred and ten suspended members; and has also sent notices to two hundred and fifty-six who are in arrears for one and two years, that unless they remit by August 10th they will not be entitled to the Transactions. The above mentioned delinquency represents, at a low estimate, $3000 due the Association. Many of the most prominent members are embraced in the list; and even two Chairmen of Sections—suspended—dropped from the roll (or will be) for non-payment of dues in three years! This shows a very great looseness in doing business. At the time these two gentlemen were elected to the Chairmanship of important Sections, they were actually not members of the Association. They were present, but under the by-laws they stood suspended at the time they were elected.

Notwithstanding the President's recommendation that the badges be placed in the hands of the Treasurer (which was done) and given out only upon the payment of dues, a very large number, and among them some of the most prominent and distinguised members, managed to get and wear badges and thereby, doubtless, got into the nominating committee, and perhaps—received office—yet failed to pay even *one* year's dues! What did we say in last issue? "Would a reputable physician, under the circumstances, take and wear a badge and yet fail to pay his dues? Wouldn't he though?" At the next meeting, our Treasurer assures us, things will be managed differently; "no dues no badge," he says, will be his motto.

Were the members to pay their dues as they should do—the Association would soon become able to reduce the yearly tax to one-half. We have 560 members; were all to pay, the yearly income, leaving out the fees from new members, would amount to $2800. As we said, about one-third of the members pay the expenses of the Association. More on this subject another time.

